# Transcriptomes define distinct subgroups of salivary gland adenoid cystic carcinoma with different driver mutations and outcomes

**DOI:** 10.18632/oncotarget.23641

**Published:** 2017-12-23

**Authors:** Candace A. Frerich, Kathryn J. Brayer, Brandon M. Painter, Huining Kang, Yoshitsugu Mitani, Adel K. El-Naggar, Scott A. Ness

**Affiliations:** ^1^ Department of Internal Medicine, University of New Mexico Health Sciences Center, Albuquerque, NM, USA; ^2^ University of New Mexico Comprehensive Cancer Center, Albuquerque, NM, USA; ^3^ Head and Neck Pathology, University of Texas MD Anderson Cancer Center, Houston, TX, USA

**Keywords:** personalized medicine, precision medicine, biomarker, emt, bioinformatics

## Abstract

The relative rarity of salivary gland adenoid cystic carcinoma (ACC) and its slow growing yet aggressive nature has complicated the development of molecular markers for patient stratification. To analyze molecular differences linked to the protracted disease course of ACC and metastases that form 5 or more years after diagnosis, detailed RNA-sequencing (RNA-seq) analysis was performed on 68 ACC tumor samples, starting with archived, formalin-fixed paraffin-embedded (FFPE) samples up to 25 years old, so that clinical outcomes were available. A statistical peak-finding approach was used to classify the tumors that expressed *MYB* or *MYBL1*, which had overlapping gene expression signatures, from a group that expressed neither oncogene and displayed a unique phenotype. Expression of *MYB* or *MYBL1* was closely correlated to the expression of the *SOX4* and *EN1* genes, suggesting that they are direct targets of Myb proteins in ACC tumors. Unsupervised hierarchical clustering identified a subgroup of approximately 20% of patients with exceptionally poor overall survival (median less than 30 months) and a unique gene expression signature resembling embryonic stem cells. The results provide a strategy for stratifying ACC patients and identifying the high-risk, poor-outcome group that are candidates for personalized therapies.

## INTRODUCTION

In an era of precision medicine, it has become increasingly important to define subgroups of patients likely to respond to specific therapeutic strategies. Adenoid cystic carcinoma (ACC), the second most frequent malignancy of the salivary glands [1], is a slow growing yet aggressive tumor with a protracted disease course typified by local recurrence and/or metastasis, which often occurs 5 or more years after diagnosis [2]. The standard treatment is surgical resection, but the effectiveness in preventing local recurrence and distant metastases is variable – survival ranges from less than 3 to more than 15 years, suggesting unexplained phenotypic or molecular heterogeneities [1, 3]. Efforts to develop targeted treatments have been largely unfruitful [4], highlighting the need for new and more effective therapeutic strategies.

ACC has been closely associated with the *MYB* oncogene since the discovery of recurrent t(6;9) translocations that fuse the *MYB* and *NFIB* genes in many of these tumors [5–7]. The *MYB* proto-oncogene encodes a DNA-binding transcription factor implicated in a variety of human hematopoietic, epithelial and neural malignancies [8–10]. The recurrent t(6;9) translocation fuses the *MYB* gene on chromosome 6 to the *NFIB* locus on chromosome 9 and may lead to overexpression of an activated Myb protein or a novel Myb-NFIB fusion oncoprotein. Detailed epigenetic studies have shown that the translocation juxtaposes important enhancers from the *NFIB* locus to the *MYB* gene, leading the oncogene to be aberrantly overexpressed [11]. However, estimates of the fraction of ACC tumors that harbor the t(6;9) translocation or that express Myb proteins or *MYB*-*NFIB* fusion transcripts have varied [1, 12–17]. These discrepancies may be due to numerous factors, including small cohort sizes, the use of frozen vs. archival FFPE material from different institutions, different types of detection methods or even problematic antibodies used in molecular assays. The confusion has led some authors to conclude that *MYB* is unlikely to be an important driver oncogene in ACC tumors [18, 19] or even that the fusion partner *NFIB* plays a more important functional role than expected [20]. These issues became even more complex with the discovery of alternative translocations in some ACC tumors. For example, instead of fusions with the *MYB* gene, a subgroup of ACC tumors display fusions of the *MYBL1* gene on chromosome 8, fused to either the *NFIB* or *RAD51B* genes [17, 21]. *MYBL1* encodes the A-Myb transcription factor that is highly related to Myb: the two proteins can bind the same DNA sequences and can activate the same target genes [9, 10]. Another subgroup of ACC tumors has been described that have point mutations in *NOTCH1* [22]. Thus, despite considerable progress, there remains uncertainty about the extent of heterogeneity amongst ACC mutations, the importance of different candidate driver oncogenes in ACC tumor development and progress and consequently what the appropriate course of action should be for developing targeted therapeutic agents.

Since metastases in ACC tumors may develop after 5 years or more, linking molecular data to outcomes is challenging due to the need to analyze relatively old samples, which may not have been preserved with RNA or DNA analysis in mind. In addition, there have been reports that several supposed ACC cell lines have been misidentified or could be contaminated by other cell types, so studies that have relied primarily on cell line analyses may be compromised [23, 24]. Fortunately, recent advances in the analysis of RNA derived from archival FFPE samples [17, 25] provide a new opportunity to analyze gene expression patterns in rare tumors like ACC, using primary patient samples that are more than a decade old. Here, we describe the unbiased RNA-seq analysis of the largest cohort of ACC tumor samples to date: 68 archival FFPE salivary gland ACC tumors accompanied by retrospective clinical data, collected over a period of 25 years. The analysis revealed unforeseen heterogeneity amongst the ACC patients and provided evidence of diverse molecular signatures amongst ACC tumors as well as genes associated with poor outcome that could serve as novel biomarkers or targets for future therapeutic strategies.

## RESULTS

### RNA-seq analysis of ACC tumor samples up to 25 years old

**Figure 1:**
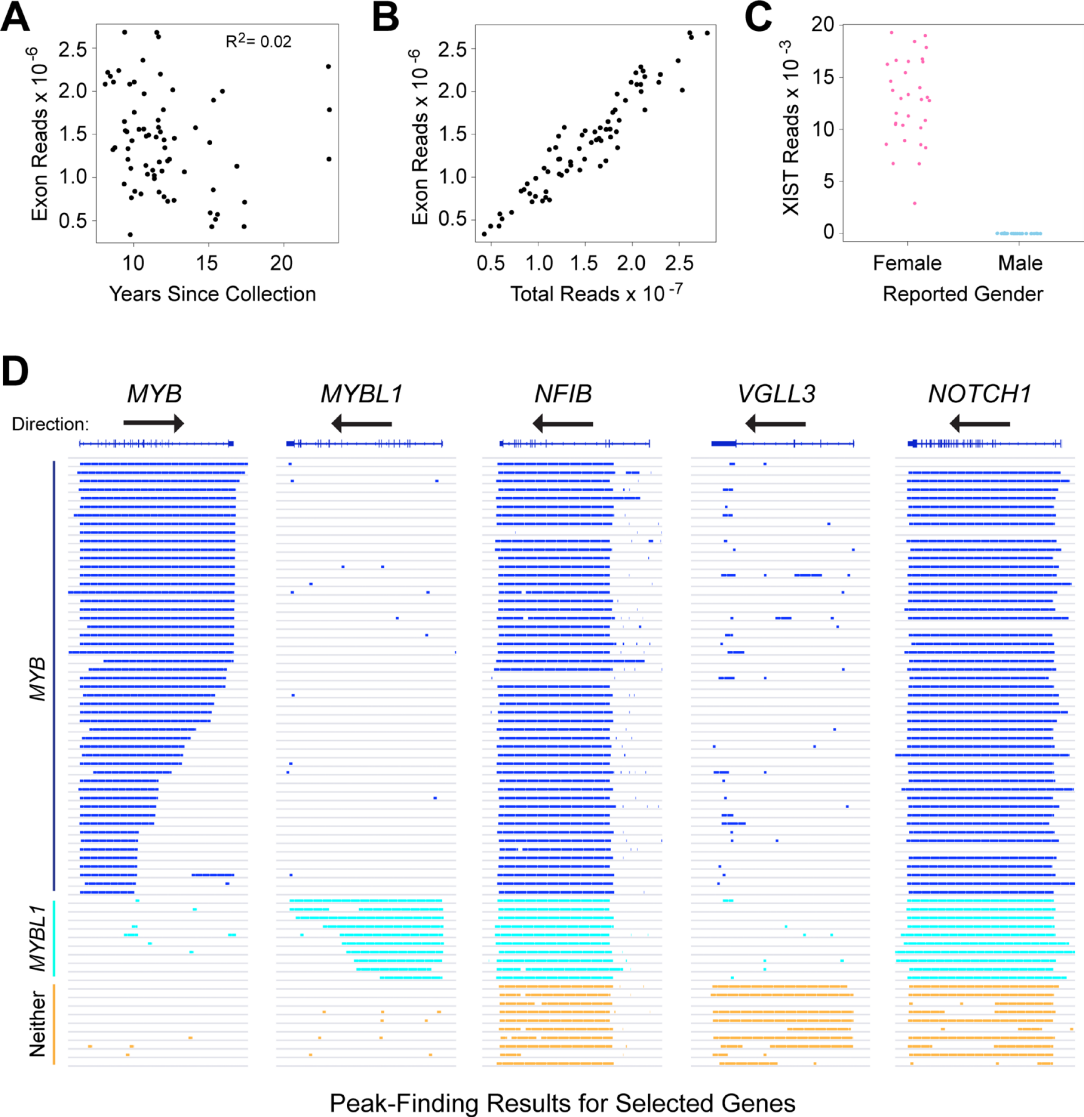
RNA-seq identifies distinct subgroups of ACC tumor samples (**A**) Plot of years since samples were collected vs. RNA-seq reads mapped to exons in the reference genome shows that high-quality results were obtained with samples collected up to 24 years ago, and that quality did not correlate with the age of the samples. (**B**) Plot of total RNA-seq reads vs. exon mapped reads, one of the quality control measures employed in this study. (**C**) Plot of reads mapped to the XIST gene as a function of reported gender in the associated clinical data. This quality control step is useful to identify mislabeled samples. (**D**) Genome browser representation of peak-calling results generated from ACC tumor sample RNA-seq data for the genes indicated. Gene names and exon/intron structures are at top, arrows show the direction of transcription, each horizontal line or track is a different ACC sample, ordered to cluster the samples with similar gene expression patterns and colored bars indicate regions of transcription detected by the peak-calling algorithm. Note that the *MYB* gene is transcribed left-to-right, but the others are right-to-left. Samples that express *MYB* (dark blue), *MYBL1* (cyan) or neither oncogene (orange) are labeled at left.

In an earlier study, we compared the RNA-seq profiles of ACC tumors to normal salivary gland, but many of those tumor samples lacked clinical follow-up data [17]. Many ACC patients survive more than 5 years after surgery before succumbing to distant metastases, necessitating the analysis of relatively old samples with informative outcome information. We tested improved RNA-seq methods [17, 25], using a small set of ACC samples collected over a range of dates up to 25 years ago. RNA was isolated from FFPE sections and analyzed using optimized library methods and the Ion Proton instrument, which has the advantage of being able to analyze fragments as short as 25 nt. More than 85% of the initial samples, regardless of their age, yielded RNA-seq data suitable for our study. We expanded our analysis to 77 samples with follow-up periods of at least 5 years, of which 68 (88%) yielded high quality RNA-seq results, with an average of ∼15 × 10^6^ reads for each sample (Table 1). An average of 9% of the reads mapped uniquely to exon features. These are all new ACC samples, not analyzed in our previous study [17]. Figure 1A summarizes the number of reads mapped to exons obtained for each sample, as a function of the years since sample collection. Although some samples performed better than others, there was not a significant correlation between the number of high quality, exon mapped reads obtained and the age of the FFPE samples (R-squared = 0.02). We also performed several types of quality control checks on the RNA-seq data and used those results to eliminate outlier samples [25]. For example, we compared the total RNA-seq reads to the exon mapped reads (Figure 1B) and the number of reads in the XIST gene, a female-specific non-coding RNA expressed from the silenced X-chromosome, as a check of the reported gender information (Figure 1C). These results confirmed that RNA-seq can provide useful gene expression information from FFPE samples, even for archived samples that were collected more than 10 years ago.

**Table 1:** RNA-seq statistics

Total Samples, *n* Female Male	68 30 38
Average Total Reads, ×10^–6^ (range) Average Exon Mapped Reads, ×10^–6^ (range)	15.17 (4.26–27.98) 1.41 (0.34–2.68)
*MYB* overexpression *MYBL1* overexpression No *MYB* or *MYBL1*	49 (72%) 7 (10%) 12 (18%)

### Most ACC tumors express either *MYB* or *MYBL1*

Although rearrangements of the *MYB* and *MYBL1* genes have been observed in many ACC tumors [17], there has been some controversy about the importance of the oncogenes [18, 19]. In addition, commercially available antibodies to measure Myb protein levels by immunohistochemistry can be problematic (data not shown), which could contribute to some of the reported differences in the fraction of ACC samples that express Myb proteins. To increase sensitivity, we started with the RNA-seq raw aligned read (e.g. ‘.bam’) files and used a peak-calling algorithm to identify the samples that did or did not express the *MYB* or *MYBL1* genes above background. The advantage of the peak-calling algorithm is that it also makes use of the reads that map to intron regions, rather than only the exon-mapped reads we used for quantifying gene expression [17, 25]. The peak-calling results for these and several other genes are shown in Figure 1D, where colored lines indicate a region of gene expression defined by the peak-calling algorithm. The results led us to divide the samples into three groups: 49 of the samples expressed *MYB* (dark blue, upper samples), 7 expressed *MYBL1* (cyan) and 12 expressed neither oncogene (orange, bottom). Overall, 56 of the 68 samples or 82% expressed either *MYB* or *MYBL1*. Interestingly, none of the samples expressed both *MYB* and *MYBL1*, consistent with the hypothesis that these are the interchangeable driver oncogenes for most ACC tumors – there is no need or selection pressure for a tumor to express both. The peak-calling algorithm was able to distinguish many of the samples in which the *MYB* or *MYBL1* genes were truncated due to translocations (indicated by shorter lines that fail to extend across the entire gene). For comparison, Figure 1D also shows the peak-calling results for several other genes: *NFIB* and *NOTCH1*, which have been implicated as important in ACC tumors and are expressed in most, but not all of the samples from all three groups, and *VGLL3*, an example of a gene that was expressed only in samples that expressed neither *MYB* nor *MYBL1*. The *VGLL3* gene encodes a transcription factor implicated in other epithelial tumors [26, 27], but its importance in ACC has not been established. We include it here as an example only to illustrate the striking differences in gene expression profiles in these samples. Two of the samples in our cohort did not express *NFIB* above background levels, suggesting that *NFIB* expression is not required for the development of all ACC tumors [20]. However, the samples that were positive expressed high levels of transcripts, suggesting that at least one allele of the NFIB gene was very highly expressed in most samples.

### Analysis of RNA-seq data for evidence of fusion transcripts

Chromosome translocations and gene fusions are important driver mutations for many types of leukemia and solid tumors, such as ACC, but their detection can be problematic. We used several approaches to attempt to identify potential gene fusions in the ACC tumor RNA-seq data. The peak-calling algorithm described in Figure 1 identified some tumors that appeared to have truncated oncogenes. A splice-aware aligning program, STAR [28], was used to identify chimeric reads that aligned to two different genes. Candidates were then verified by visually inspecting the reads using a genome browser. The results of these efforts are summarized in Table 2. Despite the relatively poor quality of the starting RNA used for these studies, and the modest read depths obtained, we were able to identify putative chimeric or fusion reads in a large fraction of the samples. We identified *MYB-NFIB* fusion reads in 11 samples and *MYBL1-NFIB* fusion reads in 3 samples. We also identified fusions between *MYB* and the *PDCD1LG2* or *EFR3A* genes in two additional samples, and validated those novel fusions by amplifying them using genomic DNA-based PCR followed by conventional (Sanger) sequencing (for details and sequencing results see Supplementary Table 1). We identified 29 samples that appeared to have truncated *MYB* gene transcripts where no fusion reads could be found, so the fusion partner remains uncharacterized. Similarly, 4 samples appeared to have truncated *MYBL1* genes based on the RNA-seq data, but insufficient fusion reads were found to identify a fusion partner. Although the analysis of RNA-seq data was able to identify many examples of fusion transcripts, this type of analysis cannot identify other types of fusions or gene rearrangements that may not lead to the expression of fusion transcripts, so the percentages of cases with translocations should be considered an underestimate.

**Table 2:** Observed and putative *MYB* gene fusions identified in 68 ACC tumors

Partner 1	Partner 2	Putative translocation^*^	No. of cases
*MYB*	*NFIB* *PDCD1LG2* *EFR3A* Fusion partner unknown	t(6;9) t(6;9) t(6;8) t(6;?)	11 1 1 29
*MYBL1*	*NFIB* Fusion partner unknown	t(8;9) t(8;?)	3 4
Tumors with apparent *MYB* truncation or translocation^*^ Tumors with apparent *MYBL1* truncation or translocation^*^ – Total number of tumors with apparent *MYB* or *MYBL1* translocations^*^ Tumors that over-express *MYB*, but no evidence of truncation Tumors that express neither *MYB* nor *MYBL1*			42 (62%) 7 (10%) – 49 (72%) 7 (10%) 12 (18%)

^*^Based only on RNA-seq results, not confirmed by FISH.

### Gene expression signatures identify major subgroups of ACC tumors

**Figure 2:**
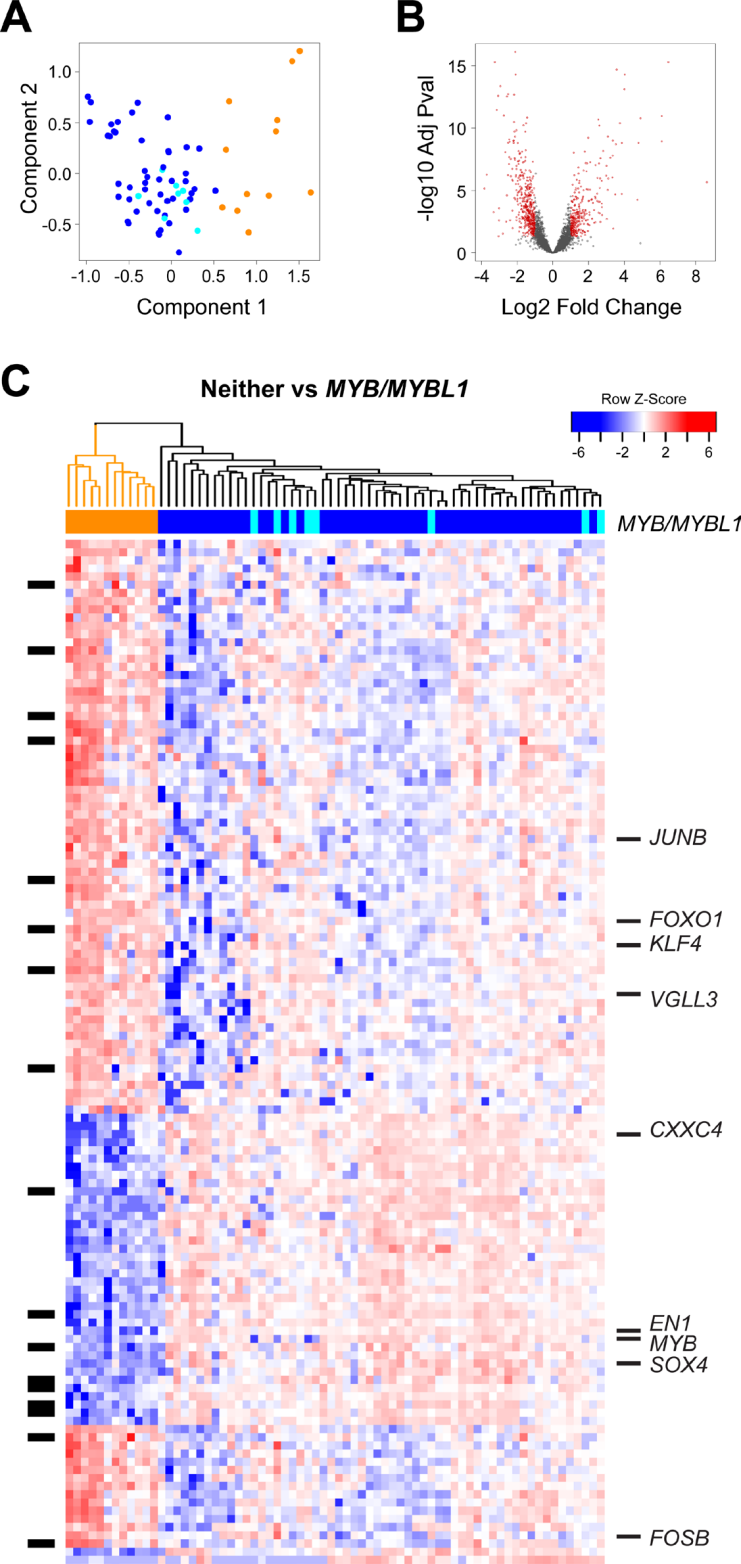
Differential gene expression analysis: *MYB*/*MYBL1* vs neither oncogene (**A**) Principal components analysis of ACC tumor RNA-seq data. The colors indicate the samples that express *MYB* (dark blue), *MYBL1* (cyan) or neither of the oncogenes (orange), as determined by the peak-calling results summarized in Figure 1D. Note that the orange samples that express neither *MYB* nor *MYBL1* separate from the others and form their own group on the right side of the plot. (**B**) Volcano plot summarizing the differential gene expression analysis, showing log2 of fold change vs. log10 of the *p*-value (BH adjusted). See Materials and Methods for details. (**C**) The heatmap summarizes the supervised clustering and differential gene expression analysis comparing the samples expressing *MYB* or *MYBL1* (marked blue or cyan at top) to the samples expressing neither oncogene (marked orange at top). The side bar at left indicates genes that are listed in the drug gene interactions database. Several interesting genes specific for the two groups are labeled at right. A larger version of this heatmap with all the genes labeled is provided in the supplementary results (Supplementary Figure 1).

In addition to the survival groups described above, our peak-calling analysis established that ACC tumors form at least three groups, based on the expression of *MYB*, *MYBL1* or neither oncogene. We characterized the gene expression signatures in the ACC tumors to investigate the differences or similarities in these groups. As shown in Figure 2A, Principal Components Analysis separated the ACC tumors into two major groups. The samples that expressed neither *MYB* nor *MYBL1* clustered at the right side of the plot (orange). The remaining tumors expressing either *MYB* (blue) or *MYBL1* (cyan) were on the left, and completely overlapped, suggesting that the two oncogenes were interchangeable and contributed to similar gene expression profiles. This is consistent with previous studies showing that swapping the DNA binding domains of the c-Myb and A-Myb transcription factors, encoded by the *MYB* and *MYBL1* genes, respectively, resulted in only minimal changes in specificity and activity [29]. The samples that expressed neither *MYB* nor *MYBL1* were specifically re-checked to make sure that they were diagnosed correctly. Re-examination revealed that all cases were adenoid cystic cancer composed of tubular and cribriform patients with no solid features. The majority arose from minor salivary gland sites (see Supplementary Table 2).

For differential gene expression analysis, we treated the tumors expressing either *MYB* or *MYBL1* (dark blue or cyan in Figure 2A) as one group and searched for genes distinguishing them from the tumors expressing neither oncogene (orange in Figure 2A). As shown in the Volcano plot in Figure 2B, our analysis identified more than 1,500 genes that were at least 2-fold up- or down-regulated, with an adjusted *p*-value of 0.05 or less. The heatmap shown in Figure 2C summarizes the supervised clustering analysis. The dendrogram and the color bar at top identify the tumors that express either *MYB* or *MYBL1* (right, dark blue and cyan) and the tumors that express neither oncogene (left, orange).

Several important conclusions can be drawn from the heatmap in Figure 2C. As described above, the tumors expressing either *MYB* or *MYBL1* do not form their own groups and do not have distinct gene expression signatures. Instead, the samples expressing *MYBL1* are scattered amongst the *MYB* samples, suggesting that the oncogenes are interchangeable and that either can suffice as the key driver for these tumors. However, there is evidence of heterogeneity amongst the tumors expressing *MYB* or *MYBL1*. Several subgroups are apparent in the dendrogram at the top, and are especially evident in the top half of the heatmap, which shows clusters of tumors with different patterns of gene expression.

Another conclusion is that there are hundreds or thousands of gene expression differences between the *MYB*/*MYBL1* samples and the tumors that express neither oncogene. This was unexpected, since all of these tumors are classified as ACC, but explains why the samples were so easily distinguished in the principal components analysis (Figure 2A). Several interesting genes have been highlighted and labeled in the heatmap in Figure 2C (a full-size version with all the genes labeled is provided as Supplementary Figure 1). Some of the most interesting genes up-regulated in the tumors that express neither *MYB* nor *MYBL1* included *JUNB*, *FOXO1, KLF4*, *VGLL3,* and *FOSB*, all of which encode transcription factors and could be potential ‘drivers’ of this ‘non-*MYB’* subgroup of ACC tumors. In contrast, the genes correlated most closely with *MYB* or *MYBL1* expression included chemokine *CXXC4* and the transcription factors *SOX4* and *EN1*. The latter gene, which encodes the engrailed homeobox 1 transcription factor, has been identified previously as an important biomarker in ACC tumors [30]. The *SOX4* gene was also identified previously as being up-regulated in ACC tumors [31]. Our results show that both *EN1* and *SOX4* are highly correlated with the expression of *MYB*/*MYBL1*, suggesting that they could be direct downstream targets regulated by the oncogenes.

### *EN1* and *SOX4* are Myb-regulated target genes in ACC tumors

**Figure 3:**
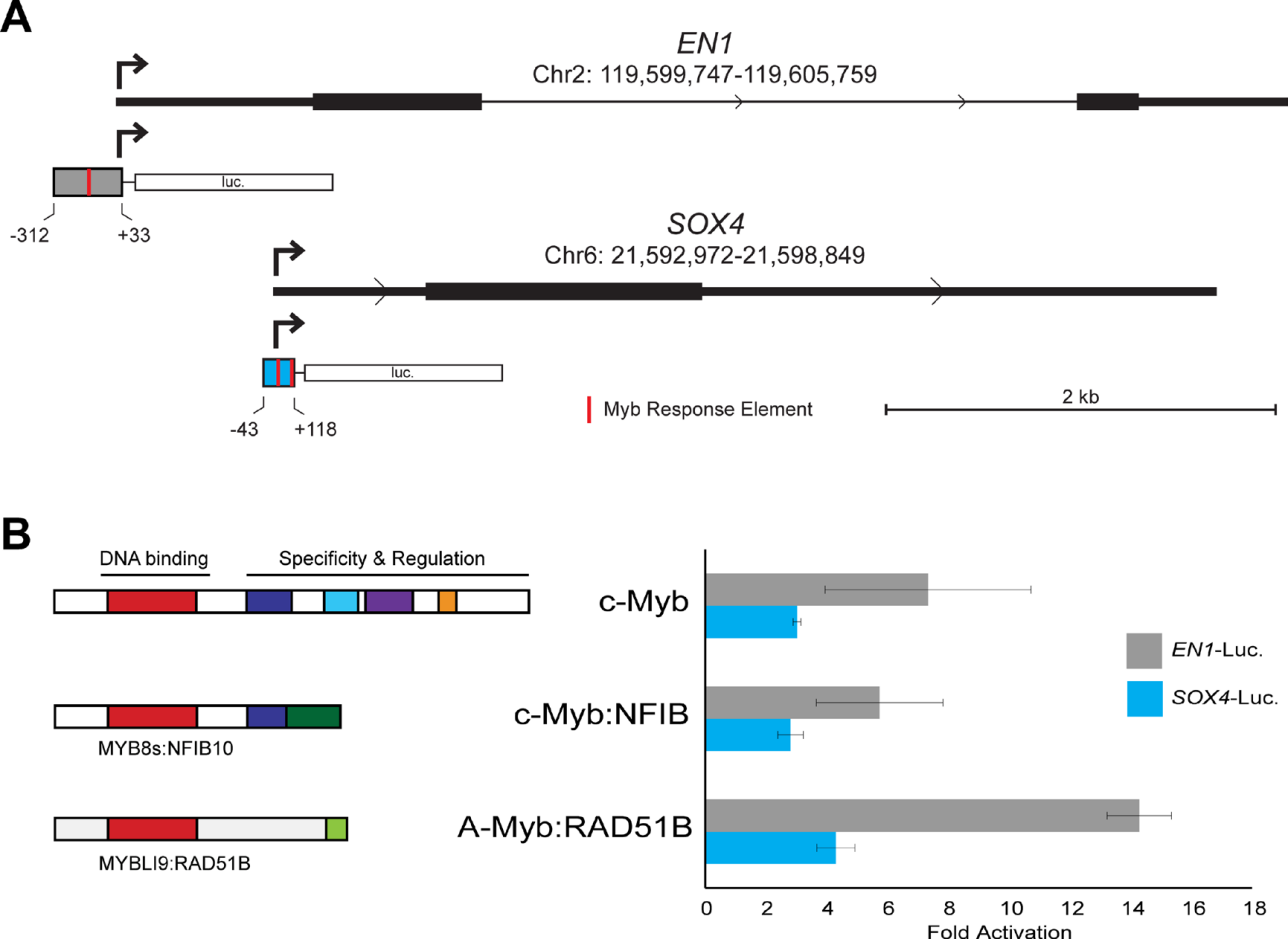
*EN1* and *SOX4* promoter reporter gene assays (**A**) Structure of *EN1* and *SOX4* promoters and reporter gene vectors. The diagrams show the 5′-end of each gene with the normal transcription start site indicated with an arrow and the fragment used for the promoter-reporter constructs indicated below. Red marks indicate predicted binding sites for Myb proteins (Myb Response Elements). The full DNA sequence of each cloned fragment is provided in Methods. (**B**) Transfection-reporter gene results. The *EN1* and *SOX4* promoter-reporter gene plasmids were co-transfected into HEK293T cells along with control (empty) vector or plasmids expressing the normal, full-length c-Myb or either *MYB*-*NFIB* or *MYBL1*-*RAD51B* fusion constructs. The diagrams at left show the structures of the fusion proteins that were expressed. The bar graph at right shows luciferase reporter gene activity normalized to the level of control (empty) vector for *EN1* (gray) and *SOX4* (blue) promoter-reporter plasmids.

Comprehensive chromatin immunoprecipitation-sequencing (ChIP-seq) results for ACC tumor samples have been reported [11]. We analyzed the publicly available data and confirmed that, although the binding is weak, both the *EN1* and *SOX4* promoters are occupied by Myb proteins in ACC tumors (data not shown). We used PCR to amplify the promoters of each gene, cloned them into reporter gene plasmids and tested their response to Myb proteins in transfection/reporter gene assays. Diagrams of the promoter regions of the *EN1* and *SOX4* genes are shown in Figure 3A, along with the regions that we cloned into the reporter gene plasmids. Both promoters contain predicted Myb Response Elements [32, 33], indicated by red marks in the promoter fragments shown in Figure 3A (the full DNA sequence of each cloned fragment is provided in the supplementary data). For functional assays, we transfected the reporter plasmids into HEK293T cells, which lack endogenous c-Myb or A-Myb protein expression, along with control (empty vector) plasmid or plasmids expressing wild type c-Myb or *MYB*-*NFIB* or *MYBL1*-*RAD51B* fusion oncogenes identified previously [17]. As shown in Figure 3B, both the *EN1* (gray) and *SOX4* (blue) reporter genes were strongly (3–14 fold) activated by co-transfection of plasmids expressing wild type or oncogenic Myb proteins. Neither promoter was significantly activated by a negative control vector expressing a c-Myb protein with a mutated DNA binding domain that is unable to bind DNA (not shown). These results confirm that both the c-Myb protein encoded by the *MYB* gene and the A-Myb protein encoded by *MYBL1* can activate the *EN1* and *SOX4* promoters in transfection assays. Based on these results, the published ChIP-seq results showing that these promoters are occupied by Myb proteins in ACC tumors, and the tight correlation between *MYB*/*MYBL1*, *EN1* and *SOX4* RNA levels in ACC tumor samples, we conclude that *EN1* and *SOX4* are likely to be direct targets of regulation by Myb proteins in ACC tumors. However, additional experiments will be required to determine whether these two Myb-regulated genes play a direct role in the development or pathogenesis of ACC tumors.

### Identification of a high-risk, poor-outcome subgroup of ACC patients

**Figure 4:**
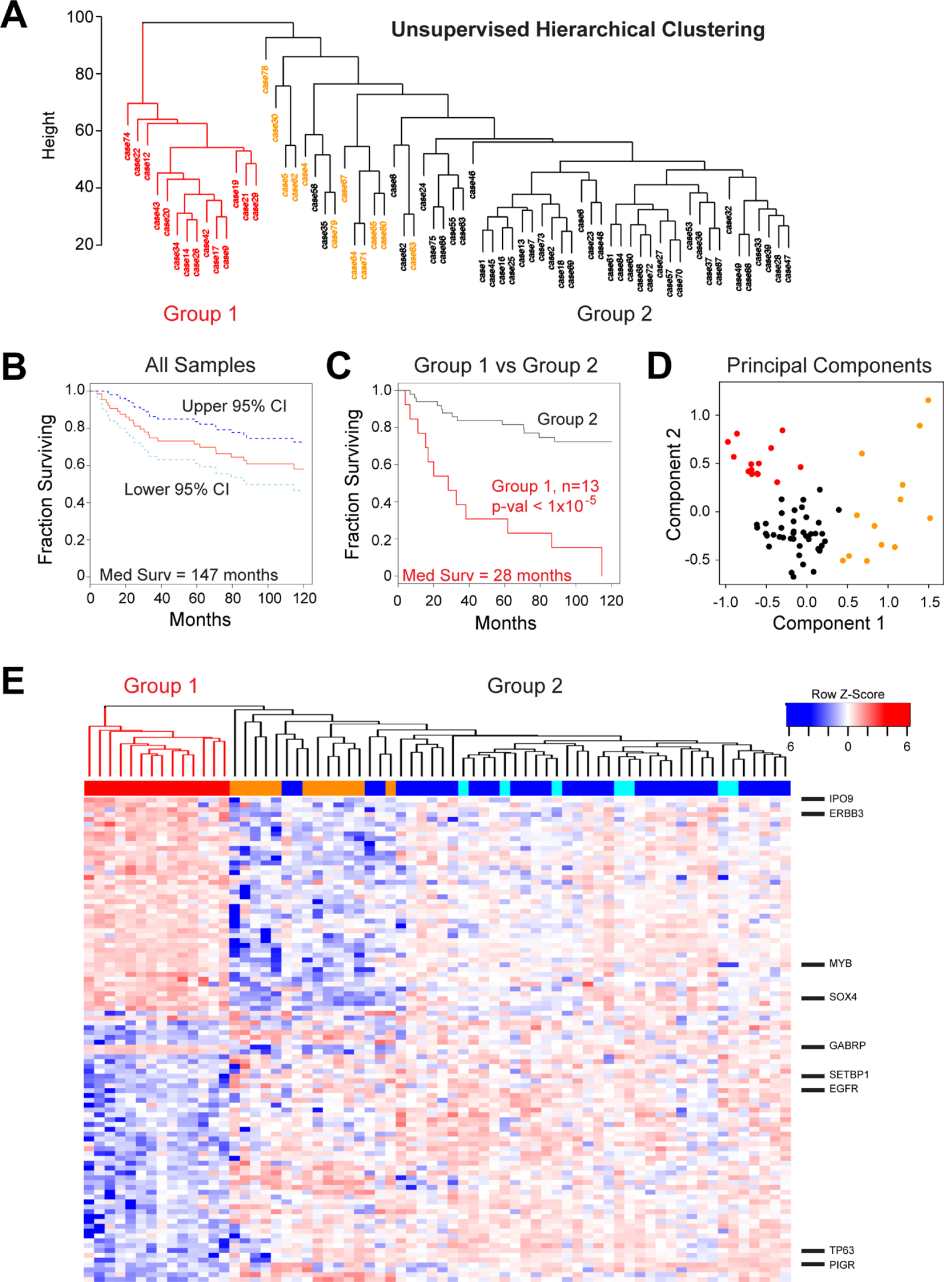
Identification of a high-risk subgroup of ACC patients (**A**) Unsupervised hierarchical clustering: ACC tumor samples form two major clusters, labeled Group 1 (red) and Group 2 (orange and black). (**B**) Kaplan–Meier survival plot for all 68 ACC tumor samples with survival information showing median survival (red) as well as 95% confidence intervals (cyan and dark blue). (**C**) Kaplan–Meier survival plots of ACC tumor samples in Groups 1 and 2 (red and black, respectively). The groups contained 13 and 55 patients, respectively. (**D**) Principal components analysis of ACC tumor RNA-seq data. The colors indicate the samples that express either *MYB* or *MYBL1* in Group 1 (red) or Group 2 (black) or the samples that express neither of the oncogenes (orange), as determined by the peak-calling results summarized in Figure 1D. Note that the samples that express neither *MYB* nor *MYBL1* (orange) separate from the others and form their own group on the right side of the plot. The poor survival Group 1 samples (red) cluster at the upper left corner of the plot. (**E**) The heatmap summarizes the results of differential gene expression analysis comparing the poor survival Group 1 (left, red) and better survival Group 2 (right) ACC samples. The color bar at top indicates samples in Group 1 (red) or samples that express *MYB* (dark blue), *MYBL1* (cyan) or neither oncogene (orange). Several interesting genes up-regulated in each group are labeled at right. A larger version of this heatmap with all the genes labeled is provided in supplementary information as Supplementary Figure 2.

ACC is a morphologically and clinically heterogeneous disease, which makes grading and treatment challenging. Our previous analyses using only 20 tumor samples suggested that there was heterogeneity amongst ACC tumors [17]. To investigate this further, we performed unsupervised hierarchical clustering of the gene expression data for the 68 new tumors, generating the dendrogram shown in Figure 4A. The ACC tumors formed two major groups. Group 1 (red, *n* = 14) was distinct and separated in the dendrogram, indicating that the samples were quite different in terms of major gene expression characteristics. Group 2 (*n* = 54, black and orange) contained the majority of cases and was composed of several smaller subgroups, implying additional genetic heterogeneity amongst ACC tumors that could be biologically important. All of the samples that expressed neither *MYB* nor *MYBL1* (orange) were in the larger Group 2. We used Kaplan–Meier survival analysis to evaluate all the samples with survival data (Figure 4B), which revealed a median survival for all ACC patients of 147 months and a 5-year survival rate of 72% (95% Confidence Interval, C.I.: 0.62–0.84). However, as shown in Figure 4C, the 13 patients in Group 1 (red) with survival information displayed a median survival of only 28 months, a mean survival of only 54% after 2 years (95% Confidence Interval, C.I.: 0.33–0.89) and a dismal 31% survival over 5 years (95% C.I.: 0.14–0.70). There were no patients in Group 1 that survived more than 10 yrs. The patients in Group 2 (black) displayed significantly better survival (log-rank *p*-value < 1 × 10^–5^) with an average 92% survival over 2 years (95% C.I.: 0.85–1.0), more than 81% survival over 5 years (95% C.I.: 0.71–0.93) and 72% survival over 10 yrs (95% C.I.: 0.61–0.87). Group 2 contained samples that expressed *MYB* plus all of the samples that expressed *MYBL1* and all of the samples that expressed neither oncogene (described in Figure 1). When tested separately, all of these subgroups had relatively good survival. The Principal Components Analysis plot in Figure 4D combines these survival clusters with the results shown above in Figure 2. The ACC samples form three distinct groups: the poor survival Group 1 samples (red) at upper left, the samples that express neither *MYB* nor *MYBL* at the right (orange), and the remainder of the *MYB* or *MYBL1* expressing samples at lower left (black). Thus, gene expression patterns identified a previously unknown subgroup of ACC patients with significantly worse overall survival and divide the ACC patients into three distinct groups with different driver oncogenes and outcomes.

A number of publications have reported markers for identifying poor survival subgroups of ACC patients [1, 30, 34–43]. Most of the markers were originally tested using antibody staining in immunohistochemistry assays, and some were developed using ACC cell lines whose authenticity have been called into question [23, 24]. We tested whether 20 previously identified markers were useful for identifying poor survival subgroups in our cohort, using the RNA-seq data. The results, summarized in Table 3, showed that only *MYB, PTK2* (FAK) and *SNAI2* (Slug) were useful for identifying subgroups of ACC tumors that showed significant differences in survival, based on using a Cox proportional hazard model analysis. None of the other markers yielded significant results, although the failures could reflect a difference between RNA expression data and protein expression as measured by immunohistochemistry assays.

**Table 3:** Genes reported to be linked to poor prognosis in ACC tumors

Gene	Reference	Correlated to	Methods^*^	HR^**^	95% C.I.		*p*-value
BMI1	[34]	Poor Outcome	IHC	0.98	0.59	1.61	0.929
CDH1	[34]	Good Outcome	IHC	1.08	0.12	9.43	0.945
EN1	[30]	Poor Outcome	IHC	2.82	0.66	12.01	0.160
EPAS1 (HIF2a)	[35]	Poor Outcome	QPCR, IHC	0.16	0.02	1.25	0.081
FABP7	[61]	Poor Outcome	QPCR	2.20	0.83	5.81	0.111
ILK	[36]	Poor Outcome	IHC	0.57	0.09	3.73	0.555
KIT	[37]	Poor Outcome	QPCR	1.13	0.17	7.42	0.897
**MYB**	**[1]**	**Poor Outcome**	**QPCR**	**3.59**	**0.95**	**13.53**	**0.059**
NOTCH1	[38]	Poor Outcome	IHC	2.11	0.08	54.54	0.653
PDCD4	[39]	Good Outcome	IHC	0.68	0.21	2.26	0.531
PIM1	[40]	Poor Outcome	IHC	0.57	0.20	1.58	0.277
PPM1D (WIP1)	[41]	Poor Outcome	IHC	1.79	0.55	5.84	0.333
PTEN	[36]	Good Outcome	IHC	0.19	0.01	3.27	0.252
**PTK2 (FAK)**	**[36]**	**Poor Outcome**	**IHC**	**126.26**	**2.89**	**5507.29**	**0.012**
SNAI1 (Snail)	[34]	Poor Outcome	IHC	0.94	0.60	1.47	0.790
**SNAI2 (Slug)**	**[34]**	**Poor Outcome**	**IHC**	**0.34**	**0.13**	**0.91**	**0.032**
SOD2	[42]	Poor Outcome	IHC	0.55	0.10	2.98	0.492
SOX2	[43]	Poor Outcome	QPCR	0.65	0.36	1.16	0.144
TWIST2	[35]	Poor Outcome	QPCR, IHC	0.85	0.50	1.43	0.529
ZEB2 (SIP1)	[35]	Poor Outcome	QPCR, IHC	0.48	0.07	3.24	0.453

^*^IHC = Immunohistochemistry; QPCR = Quantitative PCR or other PCR assay.

^**^HR = Hazard Ratio for one standard deviation increase of gene expression in log scale.

We also tested whether any of the other clinical parameters provided with our cohort of samples could be used to distinguish a poor survival subgroup. As shown in Table 4, we found that age at surgery was significantly associated with overall survival, when treated as a continuous variable (Hazard Ratio, HR = 1.42 per 10 years). The patients 50 years of age or more had a higher risk than younger patients (HR = 1.34), although the association was not statistically significant. Gender did not have a significant association with overall survival, though males had a slightly higher risk than females (HR = 1.27). As might be expected, the two clinical parameters that describe outcome, Cancer Status (either NED: No evidence of disease or with tumor) and Metastasis Status (Yes or No) were both significantly linked to poor survival. Patients with known metastases had a significantly higher risk than those without (HR = 4.86, *p*-value = 0.0008). Patients with tumor had a significantly higher risk than those with no evidence of disease (NED) (HR = 11.27, *p*-value = 0.0013).

**Table 4:** Characteristics of 68 ACC tumors

	Level	Overall	HR^*^	95% C.I.		*p*-value
**N**		68				
**Age at Surgery, mean (SD)**		50.1 (14.1)	1.42 (per 10 yrs)	1.07	1.87	0.0143
**Age Group (%)**	<50 50+	32 (47.1) 36 (52.9)	1 1.34	0.63	2.86	0.4513
**Gender (%)**	Female Male	30 (44.1) 38 (55.9)	1 1.27	0.58	2.74	0.5516
**Metastasis Status (%)**	No Yes	36 (57.1) 27 (42.9)	1 4.86	1.93	12.21	0.0008
**Cancer Status (%)**	NED With Tumor	30 (56.6) 23 (43.4)	1 11.27	2.57	49.51	0.0013
***MYB* + *MYBL1* Expression**			3.56^*^	0.71	17.91	0.123
***EN1* Expression**			2.82^*^	0.66	12.01	0.1603

Key: HR = hazard ratio; C.I. = Confidence Interval; *p*-value = *p*-value of Wald test based on Cox-regression; NED = No Evidence of Disease

^*^HR = Hazard Ratio resulted in by one standard deviation increase

We used a multivariate analysis to test whether the gene expression groups provided additional survival information compared to the other variables. As shown in Table 5, gene expression cluster Group 1 was significantly associated with poor outcome, even after adjusting for the effects of age at surgery and metastasis status (HR = 4.76, *p*-value < 0.001). Likewise, age at surgery as a continuous variable was significantly associated with worse survival after adjusting for the effects of metastasis status and gene expression cluster (HR = 1.55 per 10 yrs, *p*-value = 0.014), and patients with metastases had a significantly higher risk than those without (HR = 3.18, *p*-value = 0.027) after adjusting for the effects of age at surgery and gene expression cluster. Thus, these three variables appear to be independently associated with poor survival. Metastasis Status is an outcome marker that is evaluated years after surgery and describes the success of the treatment. In contrast, the gene expression patterns were determined from surgical samples that were collected before the outcomes were known. So information about molecular differences could be useful for predicting overall survival and for identifying patients that need different treatment strategies or who could benefit from more intensive follow-up care.

**Table 5:** Multivariate cox-regression analysis

	Level	HR	95% C.I.		*p*-value
**N**					
**Age at Surgery**		1.55^*^ (per 10 yrs)	1.10	2.20	0.014
**Metastasis Status**	No Yes	1 3.18	1.14	8.87	0.027
**Gene Expression Cluster**	Group 2 Group 1	1 4.76	1.93	11.77	<0.001

Key: HR = hazard ratio; C.I. = Confidence Interval; *p*-value = *p*-value of Wald test based on Cox-regression

^*^Hazard Ratio associated with 10-year increase in age.

### Gene expression signatures define good- and poor-outcome subgroups of ACC patients

We performed differential gene expression analysis using the groups of ACC tumors identified by hierarchical clustering, and identified over 2,000 genes that were significantly (at least 2-fold up or down, adjusted *p*-val < 0.05) different between the two survival groups. The heatmap in Figure 4E compares the expression of the 100 genes that were most significantly correlated to Group 1 (poor survival, left, color bar: red) or Group 2 (better survival, right, color bar: orange, blue or cyan). Genes up-regulated in the poor survival group included *IPO9, ERBB3, SOX4, MYB* and *GABRP*. Genes up-regulated in the better survival group included *SETBP1, EGFR, TP63* and *PIGR* (A full-sized heatmap with all the genes labeled is provided as Supplementary Figure 2). The differential expression of *ERBB3* and *EGFR* in the poor and good survival groups, respectively, suggests a difference in signaling pathways linked to epithelial to mesenchymal transition. Although WNT signature genes were not significantly enriched in our pathway analyses, several genes in the WNT signaling pathway were differentially expressed, suggesting that a WNT specific signature may be important for the differences between the good- and poor-outcome groups. However, this characterization will require additional study.

### A stem cell gene expression signature is associated with poor outcome

**Figure 5:**
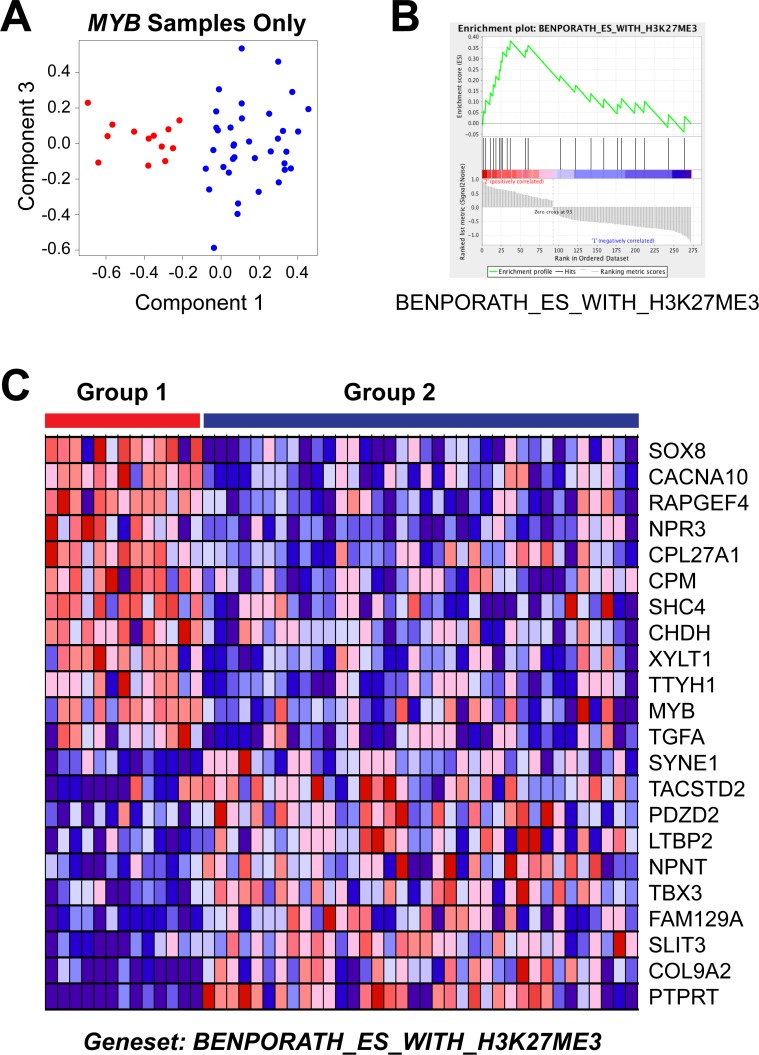
Gene set enrichment analysis of *MYB* samples (**A**) Principal Components Analysis of the *MYB* expressing samples. The colors indicate the samples in Group 1 (red, left) or Group 2 (blue, right). Note that the poor survival Group 1 samples form their own group on the left side of the plot. (**B**) Enrichment Plot of gene set ‘Benporath_ES_with_H3K27me3′ identified using the genes differentially expressed between the Group 1 and Group 2 samples expressing *MYB*. (**C**) Heatmap of differentially expressed genes from gene set ‘Benporath_ES_with_H3K27me3′, identified through Gene Set Enrichment Analysis.

We used Gene Set Enrichment Analysis [44] to illuminate the differences between the poor and good outcome subgroups of ACC tumors. A straightforward comparison of the two groups, using the differentially expressed genes for all 68 tumor samples (Figure 4E), did not identify any significantly enriched pathways, perhaps because of the dramatic heterogeneity within the good outcome group, which contained samples expressing *MYB*, *MYBL1* and neither oncogene. Therefore, since all of the Group 1 samples expressed *MYB*, we focused our analysis by comparing them only to the other samples expressing *MYB*. Principal Components Analysis (Figure 5A) cleanly separated the 13 samples in the poor outcome Group 1 (red, left) from the 36 *MYB* expressing samples in the better outcome Group 2 (blue, right). Using the approximately 1,000 genes that were significantly differentially expressed between the two groups of *MYB* expressing tumors, Gene Set Enrichment Analysis identified several gene lists that were enriched in the Group 2, good outcome samples (Table 6). However, only one gene list was significantly enriched in the poor outcome group (Table 7). As shown in Figure 5B, the genelist “BENPORATH_ES_WITH_H3K27ME3” was significantly (*p* val < 0.004) enriched in Group 1. This genelist is described as “genes possessing the trimethylated H3K27 (H3K27me3) mark in their promoters in human embryonic stem cells, as identified by ChIP on chip” [45]. This suggests that the poor outcome tumors display features related to ES cells, similar to high-grade estrogen receptor-negative, basal-like breast tumors, which also have poor clinical outcome [45]. The heatmap in Figure 5C summarizes the expression of the genes in this list in the Group 1 (left, red) and Group 2 (blue, right) tumors. The poor outcome samples are distinguished by their relatively high expression of *SOX8*, *MYB* and *TGFA* and low expression of *SYNE1*, *TBX3* and *PTPRT*. These results suggest that a gene expression biomarker could be developed to identify patients in the high-risk, poor-outcome group so they could be stratified for more intensive follow up to improve survival.

**Table 6:** Gene set enrichment analysis results: Group 2

ENRICHED IN GROUP 2 (GOOD OUTCOME)			
**GENESET NAME**	**SIZE**	**NOM p-val**	**FDR q-val**
LIM_MAMMARY_STEM_CELL_UP	41	0	0
LIU_PROSTATE_CANCER_DN	42	0	6.08E-04
SENESE_HDAC1_AND_HDAC2_TARGETS_DN	18	0	0.008256009
WANG_SMARCE1_TARGETS_UP	24	0	0.011867385
ONDER_CDH1_TARGETS_2_DN	32	0	0.013505637
MCBRYAN_PUBERTAL_TGFB1_TARGETS_UP	15	0	0.017456258
FORTSCHEGGER_PHF8_TARGETS_DN	18	0	0.022528453
ACEVEDO_FGFR1_TARGETS_IN_PROSTATE_CANCER_MODEL_DN	23	0.001287001	0.022527734
TURASHVILI_BREAST_DUCTAL_CARCINOMA_VS_DUCTAL_NORMAL_DN	29	0.001305483	0.040243994
CHANDRAN_METASTASIS_DN	15	0.001461988	0.02578634
KOINUMA_TARGETS_OF_SMAD2_OR_SMAD3	33	0.002597403	0.05427582
KINSEY_TARGETS_OF_EWSR1_FLII_FUSION_DN	22	0.004166667	0.052254837
PASINI_SUZ12_TARGETS_DN	17	0.004279601	0.034044717
MARTINEZ_RB1_AND_TP53_TARGETS_UP	15	0.005763689	0.07122304
BRUINS_UVC_RESPONSE_LATE	27	0.006648936	0.06798041
JAEGER_METASTASIS_DN	22	0.008310249	0.06001583
CHICAS_RB1_TARGETS_CONFLUENT	33	0.008782936	0.07140977

**Table 7:** Gene set enrichment analysis results: Group 1

ENRICHED IN GROUP 1 (POOR OUTCOME)			
**GENESET NAME**	**SIZE**	**NOM *p*-val**	**FDR *q*-val**
BENPORATH_ES_WITH_H3K27ME3	22	0.003861004	0.10307397

## DISCUSSION

ACC is representative of a large class of relatively rare tumors that are difficult to study because of limited samples, lack of validated cell lines and undeveloped tumor models. In the case of ACC, the disease course can be slow, often resulting in the development of metastases 5 or more years after diagnosis and surgery [2]. This necessitates the study of relatively old samples so that molecular information can be correlated to clinical outcomes. To address this issue, we developed optimized RNA-seq approaches [17, 25] so that the gene expression patterns in archived, FFPE samples up to 25 years old can be analyzed in detail. By applying these methods to one of the largest cohorts of ACC samples ever studied, we uncovered several important subsets of ACC patients and illuminated new molecular details about the oncogenic drivers that could be targeted by new types of therapeutic approaches.

We applied a novel use of a statistical peak-calling algorithm to identify the ACC samples that expressed *MYB*, *MYBL1* or neither oncogene. In addition to identifying three subgroups of ACC tumors, this approach showed that no ACC tumors in our cohort expressed both *MYB* and *MYBL1*, which is consistent with our model that the two oncogenes are interchangeable drivers of tumorigenesis [17]. With the subgroups of ACC tumors cleanly separated, we were able to perform differential gene expression analysis to identify gene expression signatures characteristic of each group. The ∼80% of tumors that expressed either *MYB* or *MYBL1* displayed dramatically different gene expression profiles than the tumors that expressed neither of the oncogenes (Figure 2). Of particular note were the *EN1* (engrailed) and *SOX4* genes that were highly correlated to the expression of *MYB*/*MYBL1*. We investigated these two genes that encode transcription factors, and found that the promoters of both genes have predicted Myb Response Elements and were activated by ectopically expressed c-Myb or A-Myb proteins in transfection-reporter gene assays. Coupled with the published ChIP-seq data showing that the promoters are occupied by Myb proteins in ACC tumors [11], we conclude that both *EN1* and *SOX4* are likely to be direct target genes activated by the *MYB/MYBL1* oncogene products in ACC tumors that express one of the oncogenes.

The other group of ACC tumors express neither *MYB* nor *MYBL1* and also do not express *EN1* or *SOX4*. Instead, they express oncogenic transcription factors *KLF4*, *FOXO1*, *JUNB* and *FOSB* and the important developmental regulator *VGLL3*. There is currently no other supporting evidence indicating that the products of these genes act as drivers of tumorigenesis in this subgroup of ACC tumors, but they seem like excellent candidates for further study and perhaps the development of animal models to test their activities.

Because of the heterogeneity we observed in the gene expression patterns in ACC tumors, we applied unsupervised hierarchical clustering to the RNA-seq results from our cohort of 68 ACC samples and unexpectedly identified a subgroup of tumors with significantly worse overall survival (Figure 4). The 68 patients that we studied included 25 who failed to survive 10 years, and more than half of those were in the poor survival subgroup identified by hierarchical clustering. The poor survival group over-expressed *ERBB3* and under-expressed *EGFR*, suggesting that poor survival may be linked to epithelial-to-mesenchymal transition or to myoepithelial vs epithelial phenotype. The poor survival samples also over-expressed *CTNNB1* and *SOX4* and under-expressed *PIK3R1* and *TP63*, all of which have been linked to tumorigenesis in other cancers. We tested 20 different markers that had been reported by others to be able to distinguish poor survival ACC tumors, but only three, *MYB*, *PTK2* (Fak) and *SNAI2* (Slug), showed significant differences in our cohort (Table 3). This probably reflects the differences in assays used–RNA levels in our assays compared to immunohistochemistry in most of the publications–but should raise a warning flag to others who wish to compare published results with studies using different technologies.

Perhaps the most important finding from these studies is the use of RNA-seq and gene expression patterns to identify a high-risk, poor-survival subgroup of ACC patients that were previously unidentified. These patients are the ones that could benefit most from increased surveillance and the development of new types of therapeutic strategies, such as targeted therapies that inactivate the *MYB* oncogene [46, 47]. The development of improved biomarkers to identify the highest-risk patients could lead to important improvements in the treatment of ACC tumors.

## METHODS

### Human salivary gland ACC FFPE samples

De-identified salivary gland adenoid cystic carcinoma FFPE tumor samples were obtained from the Salivary Gland Tumor Biorepository (MD Anderson Cancer Center, Houston, TX, USA; Table 1). A search of MD Anderson Head and Neck tumor bank for salivary gland adenoid cystic carcinoma led to the identification of 100 patients with primary section at our institution. Hematoxylin and eosin stained slides of all tumors were retrieved and were subjected to an independent, blinded review by two specialized head and neck pathologists. The phenotypic assessment of ACC was strictly made on the histopathologic finding of tubular and cribriform patterns with dual cellular formation and light luminal polysaccharide secretion with and without solid component. Tumor with solid form lacking tubular/cribriform foci were not included. The review confirmed the diagnosis of ACC in all tumors. Due to the long disease course for ACC tumors, samples were chosen that had at least 5 yr follow-up. All samples were collected with informed consent of the donors, and studies were conducted in accordance with the principle of the Declaration of Helsinki. All studies were performed with Institutional Review Board-approved protocols.

### RNA isolation and sequencing

Total RNA was isolated from one or two 5-micron slide-mounted FFPE sections using the RNeasy FFPE kit (Qiagen). cDNA synthesis and library preparation were performed using the SMARTer Universal Low Input RNA Kit for Sequencing (Clontech) and the Ion Plus Fragment Library Kit (Life Technologies) as previously described [17]. Sequencing was performed using the Ion Proton and Ion S5/XL systems (Life Technologies) in the Analytical and Translational Genomics Shared Resource at the University of New Mexico Comprehensive Cancer Center. RNA sequencing data is available for download from the NCBI BioProject database using study accession number PRJNA287156.

### Data analysis

Low quality and non-human RNA-seq reads were identified and removed from the analysis pipeline using the Kraken suite of quality control tools [48, 49]. High-quality, trimmed, human RNA-seq reads were aligned to the human genome (GRCh37; hg19) using TMAP (v5.0.7) and gene counts were calculated using HT-Seq as previously described [17]. Poor quality samples, defined as those samples which had fewer than 10% of the median number of reads of all samples, were removed from further analyses. Several additional samples were removed based on the quality control measures described in the text. Peak finding to identify samples that expressed *MYB* or *MYBL1* was performed using *findpeaks* from the HOMER (v4.9) package [50], with settings of -region, -size 1000 and -minDist 10000.

### Unsupervised hierarchical clustering

Analyses were limited to genes that were highly expressed above a threshold level in a number of samples (e.g. 250 reads in at least 10 samples). Hierarchical clustering was performed on an expression matrix of 882 highly expressed genes in 68 ACC tumors with the Euclidean distance as the dissimilarity measure and the default “complete” clustering method from the *hclust* command in the package *stats*, part of statistical software R/Bioconductor [51]. Genes that correlated with molecular subgroup were discovered using the samr R package [52, 53] to identify genes positively and negatively correlated with the first and second dimensions of a PCA plot describing these data [54].

### Statistical analysis

The primary endpoint for the outcome was overall survival (OS), defined as time from the date of diagnosis to the date of death. Subjects who were lost to follow-up or alive within the follow-up period were censored at the date of the last contact. The OS was estimated using the Kaplan–Meier method, and the differences in OS were examined using the log-rank test. Univariate Cox regression was used to assess the associations of clinical characteristics or genomic features with survival outcome, and multivariate Cox regression was used to compare amongst these variables. All the statistical analyses were performed using statistical software R (54).

### Translocation verification

For detection of putative fusions, samples with apparent *MYB* or *MYBL1* translocations, evidenced by a lack of reads mapped to the 3′ end of the gene, were examined for chimeric reads containing sequence matching the *MYB* or *MYBL1* gene and another gene. Chimeric reads were detected using the Integrative Genomics Viewer (version 2.3.79) [55, 56] with “show soft clips” turned on, and then secondarily aligned to GRCh37/hg19 using BLAT at the UCSC genome browser [57, 58] to determine the translocation partner. Samples that had obvious truncations but for which no chimeric reads were identified, were categorized as “unknown”. Novel translocations were verified by RT-PCR amplification of RNA isolated from FFPE slices as previously described [17]. Gene-specific primers used to amplify cDNA and resulting Sanger sequences are provided in Supplementary Table 1.

### EN1 and SOX4 promoter fragments

Fragments of the human EN1 and SOX4 gene promoters were isolated by PCR amplification from genomic DNA and cloned into the pGL3-basic reporter backbone. A 345 bp fragment of the EN1 promoter was amplified (forward: 5′-GAGGAGGAGCTCGAGAACGTAAACTGTCGACGC, reverse: 5′-GAGGAGGAGAAGCTTAGAGAAATGCAGGATTATGGGTC) and cloned using XhoI and HindIII restriction sites included in the primers. Similarly, a 161 bp fragment of the SOX4 promoter was amplified (forward: 5′- GAGGAGGAGGCTAGCTGCAGCCAAGACTGTGAAAG, reverse: 5′-GAGGAGGAGCTCGAGAGGAGTTCCTCCAGTGCAGA) and cloned using XhoI and NheI restriction sites. Insert sequences were verified via conventional (Sanger) sequencing.

Insert sequences were verified via conventional (Sanger) sequencing. Underlined portions are untranslated regions. Predicted Myb binding motifs are in bold+underlined. Genomic coordinates (hg19) of the inserts are shown. The EN1 promoter fragment that we cloned contains a common polymorphism (rs3731613) compared to the reference hg19 sequence, indicated by a lower case ‘g’.

**>SOX4-luc chr6: 21593929-21594089**

GCTGCAGCCAAGACTGTGAAAGGATAAAGAGGCGCGAGGCGGAATTGGGGTCTGCTCTAAGCTGCAGCAAGAG**AAACTGTGTGT**GAGGGGAAGAGGCCTGTTTCGCTGTCGGGTCTCTAGTTCTTGCACGCTCTTTAAGAGTCTGCACTGGAGGAACTCCT

**>EN1-luc chr2: 119605727-119606071**

AACGTAAACGTGCGACGCTAGCTAGGCGCAGCGGGCCTTTCAGATTTTGCTATTTGTGAAAAACAAATTGCGCCTCTGAAAGTAACCAACTCTAGGTCTATTTCACATCACCGACCTCCCTGTCTCACTCCCCCTCCCTCCACTACACACACCCAAACCCACACCCACCCACAAACACAC**AAACCGGCAG**TGACAACAACCACCCATCCTTCAATAACAGCAACCAGAGACAGAGGAGAAAATAAAAAGCTGAGTTTCTTAGGCGTGGGGGTGCAAAACAGCCAGGCTCCTGCCTACTGCCCCTGCTCCCGgAGCTCACAGACCCATAATCCTGCATTTCTCTAA

### Transfections and reporter gene assays

*MYB* and *MYBL1* fusion expression vectors, cloned into pCDNA3, were described previously [17]. Specifically the c-Myb:NFIB fusion contains a cDNA fragment spanning exons 1–8s [59, 60] of *MYB* (NM_001130173) encoding the first 313 aa of c-Myb fused to exons 10–11 of *NFIB* (NM_001282787), which adds 73 novel amino acid residues to the truncated Myb protein. The A-Myb:RAD51B fusion contains exons 1–9 of *MYBL1* (NM_001080416) encoding the first 367 aa of A-Myb fused to an intronic region of *RAD51B*, which adds 28 novel amino acids to the truncated A-Myb protein. Reporter gene assays were performed in HEK293T cells in triplicate. Cells were seeded at approximately 4 to 6 × 10^4^ cells per well in a 24 well plate and allowed 24 hours growth before transient co-transfection with 50 ng of luciferase reporter plasmid (EN1-luc or SOX4-luc in pGL3-basic) and 5 ng of activator plasmid (*MYB* or *MYBL1* fusions cloned into pCDNA3). Transfections were performed in duplicate using the TransIT-2020 transfection (Mirus) reagent according to the manufacturer’s instructions. Cells were harvested and luminescence was measured after 48 hours using the Luciferase Assay System (Promega). Background subtracted data were normalized to cells transfected with the reporter plasmid and no activator (empty pCDNA3). All experiments were performed at least in triplicate.

## SUPPLEMENTARY MATERIALS FIGURES AND TABLES



## Abbreviations

ACC: Adenoid cystic carcinoma
FFPE: Formalin-fixed, paraffin-embedded
RNA-seq: Ribonucleic acid sequencing
FISH: Fluorescence In Situ Hybridization
