# Supplementary material for: Transcriptomes define distinct subgroups of salivary gland adenoid cystic carcinoma with different driver mutations and outcomes
Source: Oncotarget. 2017 Dec 23;9(7):7341–58. doi: 10.18632/oncotarget.23641 (PMC5800907; doi:10.18632/oncotarget.23641)
Supplement: Supplementary file 1 [file oncotarget-09-7341-s001.pdf]

Transcriptomes define distinct subgroups of salivary gland adenoid cystic carcinoma with different driver mutations and outcomes

SUPPLEMENTARY MATERIALS

Supplementary Table 1: Sanger sequencing confirmation of fusions

Tumor	case9	case72
**Fusion**	MYB-PDCD1LG2	MYB-EFR3A
**Primer 1 Name**	CM6F	CM12F
**Primer 1 Target**	MYB exon 6	MYB exon 12
**Primer 1 Sequence**	CTACAATGCGTCGGAAGGTC	GCAGGATGTGATCAAACAGG
**Primer 2 Name**	PDCD1LG2revB	EFR3Aexon23rv
**Primer 2 Target**	PDCD1LG2 intron 3	EFR3A exon 23
**Primer 2 Sequence**	CCCAAACCTTGCTGATTCTT	TCAGTACACACACAGATCTGGAA
**PCR Product Sequence**	GGAGAAATACCTTCCCTTCTCCCTCCA GTCCACCCTCTAGTGTTCCTGTCTTGCT TCCTATTGGCTGTCACGTTCCTAGGCTAT ACAGGCCACAGGAGTCTGCCTTCCTGC AAGGGGCTCGCCAGGGACCTGTTTTTA GGTACTGTAAATGCTTTGAGAACATTGT CTTCATCTTCTGATGCTGGTGCCATTAAA	TGCAGGATGTGATCAAACAGGAATCTG ATGAATCTGGAATTGTTGCTGAGTTTCA AGAAAATGGACCACCCTTACTGAAGAA AATCAAACAAGAGTCCTCCTCCCAGTCC ATCAGGAACACTGACCATTACTTCTGGG CATGCCCAATACCAATCTGTCCCAGTCTAT GAGATGAAGTTTCCAGATCTGTGTGTGTACT

Supplementary Table 2: Pathology characteristics of "Neither" group samples

De-Identifier	GROUP_NAME	SITE	DX	Pattern
Case 4	Neither	palate, NOS	adenoid cystic carcinoma	Tubular/Cribriform
Case 5	Neither	tongue, NOS	adenoid cystic carcinoma	Tubular/Cribriform
Case 30	Neither	trachea	adenoid cystic carcinoma	Tubular/Cribriform
Case 62	Neither	lymph node, neck	adenoid cystic carcinoma	Tubular/Cribriform
Case 64	Neither	orbital	adenoid cystic carcinoma	Tubular/Cribriform
Case 65	Neither	maxilla	adenoid cystic carcinoma	Tubular/Cribriform
Case 67	Neither	tongue, NOS	adenoid cystic carcinoma	Tubular/Cribriform
Case 71	Neither	parotid	adenoid cystic carcinoma	Tubular/Cribriform
Case 78	Neither	parotid	adenoid cystic carcinoma	Tubular/Cribriform
Case 79	Neither	orbital	adenoid cystic carcinoma	Tubular/Cribriform
Case 80	Neither	nose	adenoid cystic carcinoma	Tubular/Cribriform
Case 83	Neither	mandible	adenoid cystic carcinoma	Tubular/Cribriform

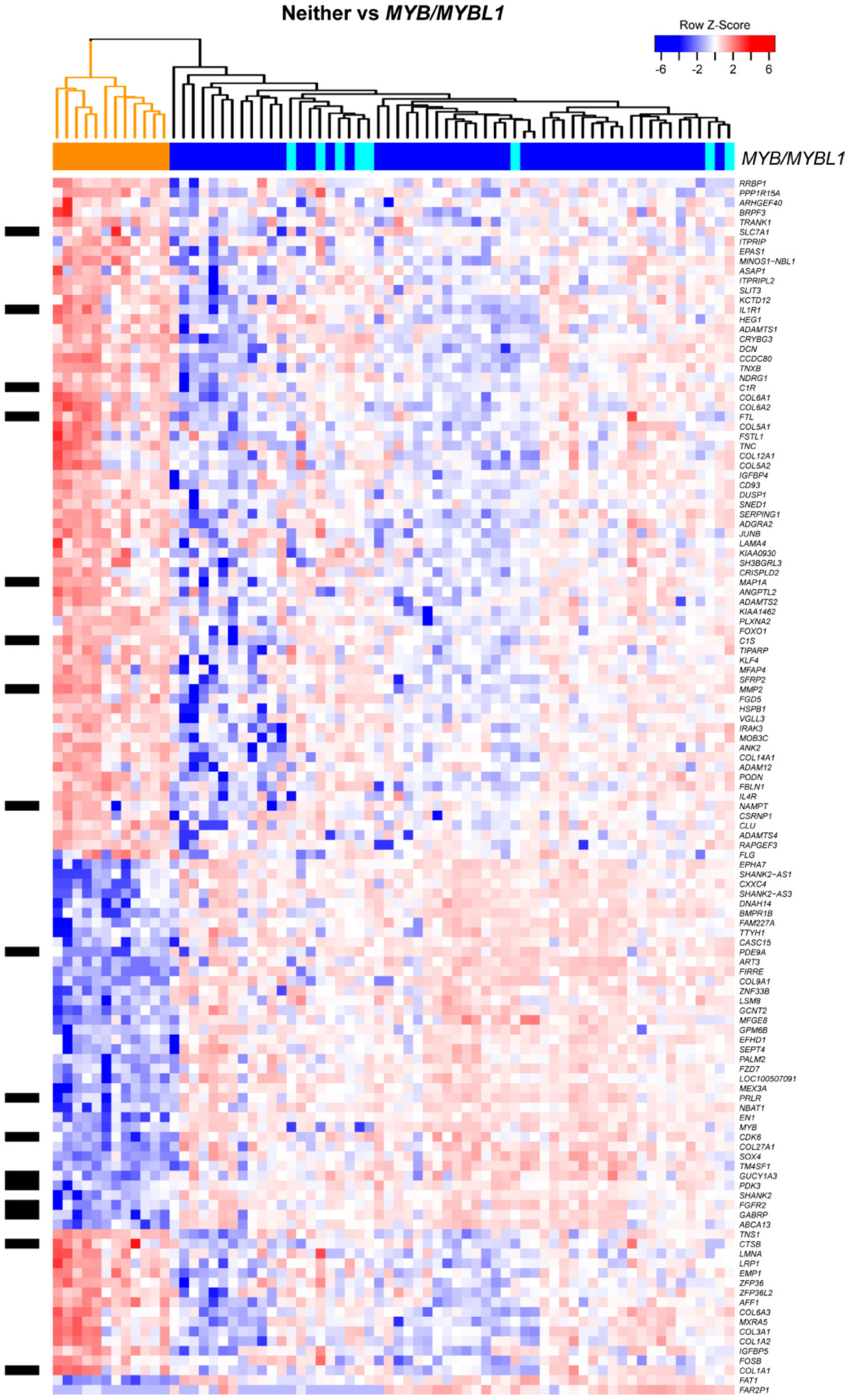

Supplementary Figure 1: Differential gene expression analysis: *MYB*/*MYBL1* vs neither oncogene. The heatmap summarizes the differential gene expression analysis comparing the samples expressing *MYB* or *MYBL1* (marked blue or cyan at top) to the samples expressing neither oncogene (marked orange at top). The side bar at left indicates genes that are listed in the drug gene interactions database. Several interesting genes specific for the two groups are labeled at right. This is a larger version of the heatmap shown in Figure 2C.

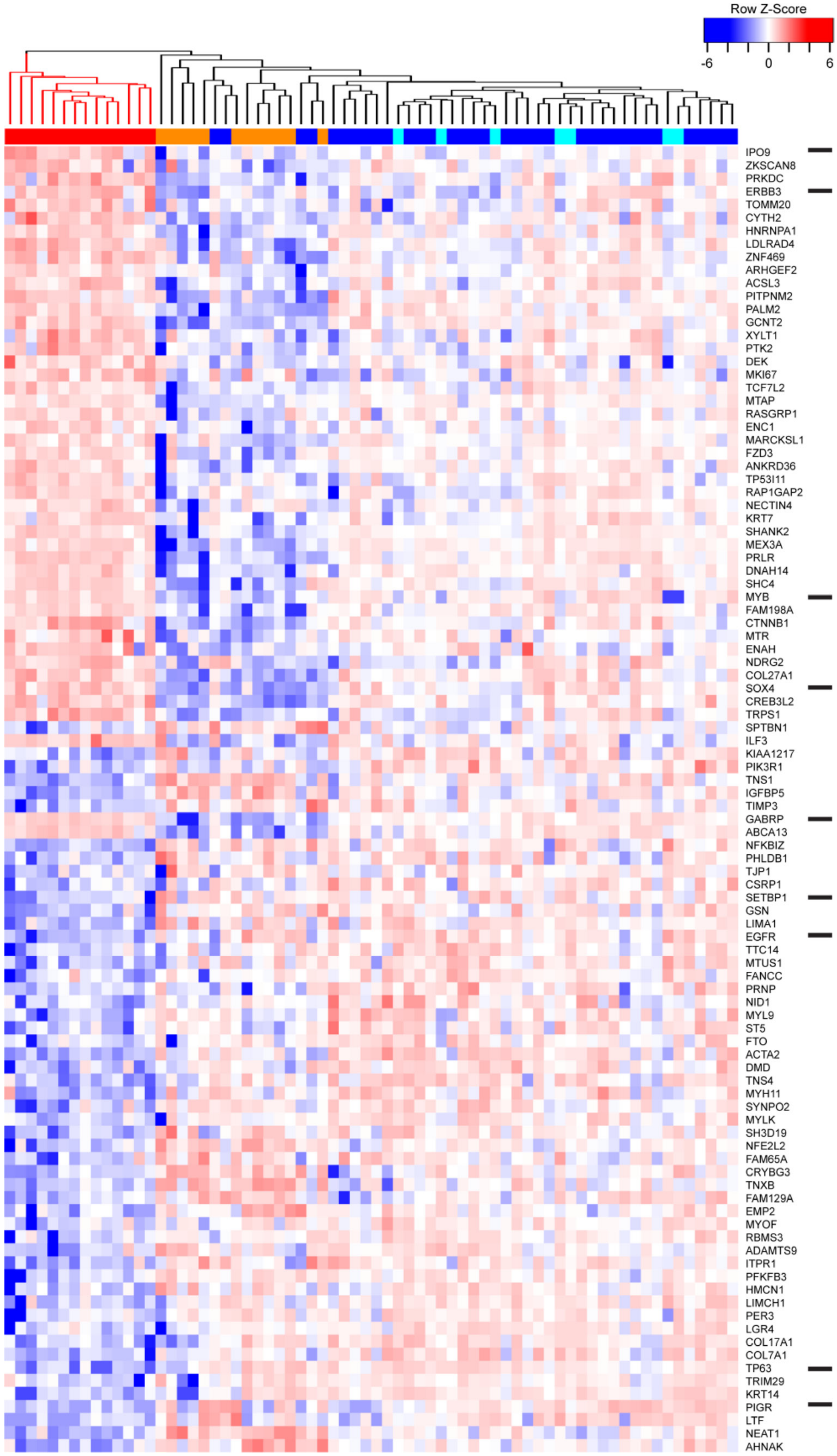

Supplementary Figure 2: Gene expression profiles correlated with poor survival. The heatmap summarizes the results of differential gene expression analysis comparing the poor survival Group 1 (left, red, *n* = 14) and better survival Group 2 (right, gray, *n* = 54) ACC samples. The color bar at top indicates survival group as identified in Figure 4. Several interesting genes up-regulated in each group are labeled at right. This is a larger version of the heatmap shown in Figure 4E.
